# Leukocytoclastic Vasculitis and Myositis as Initial Manifestations of Crohn’s Disease

**DOI:** 10.7759/cureus.33127

**Published:** 2022-12-30

**Authors:** Beatrice E Torere, Henry O Aiwuyo, Jennifer L Kennard

**Affiliations:** 1 Internal Medicine, North Mississippi Medical Center, Tupelo, USA; 2 Internal Medicine, Brookdale University Hospital Medical Center, Brooklyn, USA; 3 Rheumatology, North Mississippi Medical Center, Tupelo, USA

**Keywords:** inflammatory bowel disease, extra-intestinal manifestations of crohn's disease, myositis, leukocytoclastic vasculitis (lcv), crohn’s disease (cd)

## Abstract

Crohn’s disease is a chronic inflammatory condition that mainly affects the digestive tract; however, it possesses extra-intestinal manifestations. We present a case of a 19-year-old male with a history of non-specific gastrointestinal (GI) symptoms of nausea, vomiting, and diarrhea who underwent a colonoscopy with a biopsy due to worsening GI symptoms. The colonoscopy was inconclusive for GI pathology. Three months later, he developed several symptoms, which were later indicative of leukocytoclastic vasculitis and myositis as extra-intestinal manifestations of Crohn’s disease. The patient was started on high-dose prednisolone, which improved his symptoms remarkably, and the steroid dose was tapered gradually. He was subsequently followed up by the Rheumatology and Gastroenterology outpatient departments. The case reinforces the need for physicians to have a high index of suspicion in patients with non-specific GI symptoms presenting with new-onset cutaneous manifestations and myositis.

## Introduction

Crohn’s disease is an inflammatory bowel disease involving the gastrointestinal (GI) tract, from the mouth to the anus, with the terminal ileum being the most common part of the gut that is involved in its pathogenesis [1]. It usually progresses to chronic inflammation with inflammatory cells that can lead to transmural inflammation of any part of the GI tract [2]. The cause of this condition is unknown; however, an auto-immune etiology has been suggested to be the cause [1-3]. The disease follows a relapsing and remitting course with flares that may warrant hospitalization and negatively impact the patient's quality of life [3].

Patients affected by this condition present in a myriad of ways. GI symptoms range from diarrhea, nausea, vomiting, and abdominal pains, to bleeding per rectum [1]. Some patients present with systemic symptoms, including weight loss, fever, easy fatigability, and malaise [1,3]. Some risk factors for disease progression include smoking, young age, peri-anal and rectal involvement, and colonic disease [1,4]. It affects the small bowels in many cases, especially the terminal ileum, followed by a combined involvement of the ileum and colon [1]. Advanced disease can be complicated by intestinal perforation, strictures, fistula, abscess formation, bowel obstruction, and malignancy, especially when the colon is involved [1-3].

Notably, extra-intestinal manifestations have been reported in patients with Crohn’s disease, and it is primarily related to disease activity and chronicity [5,6]. The extra-intestinal manifestations include ocular, musculoskeletal, cardiac, vascular, and hepatobiliary systems [1,5-7]. However, this extra-intestinal presentation may precede the event of overt intestinal Crohn’s disease. We present a case of a young Caucasian teenager whose initial presentation of Crohn’s disease was leukocytoclastic vasculitis (LCV) and myositis.

## Case presentation

Our patient is a 19-year-old male Caucasian who developed an insidious onset of non-specific GI symptoms. He was well a year prior to the presentation when he developed nausea, vomiting, diarrhea, and lower abdominal pains. His symptoms worsened and warranted referral to a gastroenterologist. He was reviewed, and a colonoscopy was performed, which was inconclusive for any colonic pathology. Esophageal-gastro-duodenoscopy (EGD) revealed gastritis of the gastric fundus and antrum and erythematous duodenopathy, biopsies were taken with cold forceps, and the campylobacter-like test (CLOtest) was negative for *Helicobacter pylori*. Duodenum biopsy showed acute duodenitis with no evidence of celiac sprue, dysplasia, malignancy, and granulomas. He was eventually treated empirically for duodenitis and gastritis. Two months later, he presented to the GI department with complaints of progressive abdominal pain. He described the abdominal pain as lower abdominal “cramps,” intermittent, 7/10 in intensity, two to five episodes daily, with each episode lasting about 30 minutes to one hour, non-radiating, with associated nausea and two to three episodes of daily non-bloody diarrhea. The pain is worse with movement, with no alleviating factor. Physical examination was remarkable for lower abdominal tenderness with no rebound or guarding. The celiac disease panel was negative. A colonoscopy was performed, which revealed a normal entire colon; no ulcers, erythema, or other lesions were seen on examination. Biopsies for histology were taken. The colon biopsy revealed colonic mucosa with no significant histopathologic abnormality. No evidence of microscopic colitis was identified. The patient was treated symptomatically for gastritis. Four months after the initial presentation, he developed non-palpable petechiae rashes on his lower extremities (LE) and was subsequently seen by a dermatologist who made an impression of LCV by physical examination. He continued having similar intermittent lower abdominal discomfort accompanied by diarrhea, nausea, anorexia, and weight loss. A repeat EGD showed a normal esophagus, normal stomach, and normal duodenum. He continued outpatient follow-up with the gastroenterologist while having relapsing and remitting GI symptoms. Five months later, he presented to his primary care provider with complaints of progressive muscle weakness and pains affecting the LE about 3-4 hours after working out. After a routine workout, he will have to use his arms to pull himself up a few hours later. He reported accompanying pain in his entire thighs, which usually starts at the feet and progresses up to the thighs, with associated LE joint stiffness lasting 30 minutes to one hour. He ingested protein powders daily with no improvement in his symptoms. Subsequent evaluation revealed a positive antinuclear antibody (ANA), elevated C-reactive protein, and elevated creatine kinase (CK) concerning for myositis; he was referred to the rheumatology service. Rheumatology examination was significant for faint LE petechiae rashes. No heliotrope rash or Gottron’s papules were noted, and muscle strength was 4/5 in bilateral proximal LE muscle groups, otherwise 5/5 muscle strength throughout. Autoimmune myositis panel, extractable nuclear antibodies, antineutrophil cytoplasmic antibodies (ANCA), complements 3 and 4, and rheumatoid factor screening were all negative (Table 1).

**Table 1:** Laboratory findings of the patient WBC, white blood cell; HBsAg, hepatitis B surface antigen; Ab, antibody; HCV, hepatitis C virus; TB, tuberculosis; CRP, C-reactive protein; ESR, erythrocyte sedimentation rate; IgG, immunoglobulin G; IgA, immunoglobulin A; SM, smooth muscle; RNP, ribonucleoprotein; SS-A, SS-B, SCL-70, types of antibodies, JO-1, antihistidyl-tRNA synthetase; PL, alanyl; EJ, glycyl; OJ, isoleucyl; SRP, signal recognition particle; MI-2, Mi-2/nucleosome remodeling; MDA-5, melanoma-differentiation associated gene 5; TIF-1Y, transcriptional intermediary factor 1-gamma; NXP, nuclear matrix protein; IgG, immunoglobulin G; IgA, Immunoglobulin A; ↑, findings above normal limit; ↔, findings within normal limit

Test	Finding	Reference range
Hemoglobin (g/dL)	13.9 ↔	11.4–15.5
Platelets (uL^-1^)	226 ↔	180–400
WBC (uL^-1^)	5.5 ↔	4.2–10.2
HBsAg and core Ab	Negative	
HCV Ab	Negative	
TB gold	Negative	
Creatine kinase (IU/L)	241 ↑	55–170
CRP (mg/dL)	1.8 ↑	<1
ESR (mm/hr)	11 ↔	0–15
Aldolase (U/L)	8.1 ↔	8.1
Serum myoglobin (ng/mL)	29.6 ↔	0–121
Complement C3 (mg/dL)	127 ↔	82–185
Complement C4 (mg/dL)	25 ↔	15–53
Myeloperoxidase Ab (AI)	<1 ↔	< 1
Gliadin IgG (Eu)	3 ↔	<20
Gliadin IgA (Eu)	19 ↔	<20
Quest (IGA)	373 ↑	40-310 mg/dL
Tissue transglutaminase (IGA)	1 ↔	<4
Endomysial antibody (IGA)	Negative	
Sjogren’s Ab (SS-A)	<1.0 ↔	<1.0
Sjogren’s Ab (SS-B)	<1.0 ↔	<1.0
SM Ab	<1.0 ↔	<1.0
RNP Ab	<1.0 ↔	<1.0
SCL-70 Ab	<1.0 ↔	<1.0
JO-1 Ab	<1.0 ↔	<1.0
Anti-DNA antibody, double-stranded (IU/mL)	≤ 4	≤ 4 = Negative; 5-9 = Indeterminate; ≥ 10 = Positive
Proteinase 3 Ab serum	< 1.0	< 1.0
Anti-JO1 (SI)	< 11	< 11
PL-7 (SI)	< 11	< 11
PL-12 (SI)	< 11	< 11
EJ (SI)	< 11	< 11
OJ (SI)	< 11	< 11
SRP Ab (SI)	< 11	< 11
MI-2 alpha Ab (SI)	< 11	< 11
MI-2 beta Ab (SI)	< 11	< 11
MDA-5 (SI)	< 11	< 11
TIF-1Y Ab (SI)	< 11	< 11
NXP-2 (SI)	< 11	< 11

**Figure 1:**
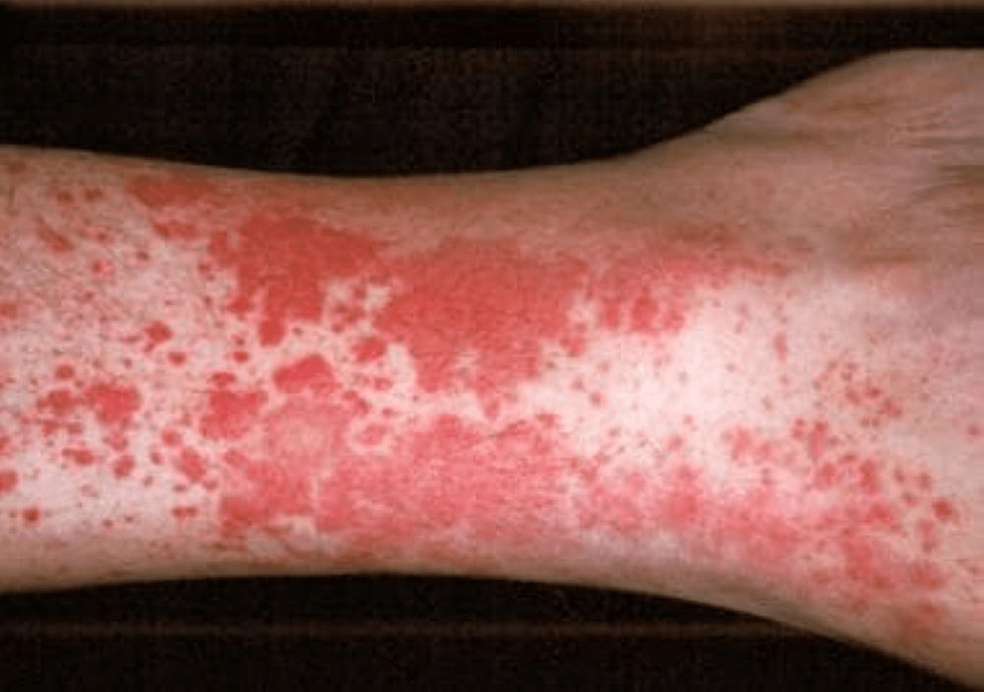
Non-palpable purpuric rash in leukocytoclastic vasculitis Image reproduced from Eastham [7].

**Figure 2:**
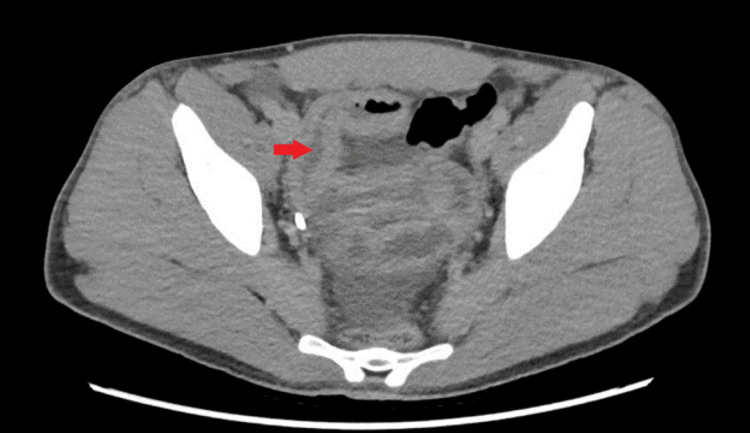
CT of the abdomen/pelvis showing inflammation of the terminal ileum Arrow indicates inflammation/thickened bowel wall of the terminal ileum/colon

Also, the purified protein derivative test, interferon test, and hepatitis panel were all negative, and the liver function test showed that aspartate and alanine transaminases were within normal limits. Creatinine kinase was elevated. Following the laboratory findings, he was started empirically on prednisolone 60 mg. Two months after, he presented to the emergency department (ED) with rectal bleeding, nausea, vomiting, diarrhea, and lower abdominal pains. Significant examination findings were lower abdominal tenderness on palpation and petechiae rash in the LE (Figure 1). On ED arrival, the patient was febrile, and the laboratory investigation showed a white blood cell count of 17,000 per microliter. He also underwent a computed tomography (CT) scan of the abdomen and pelvis, which showed infectious/inflammatory enteritis involving the distal segment of small bowel loops with mild adjacent ascites, distended appendix containing fluid and air, but no supportive evidence of appendicitis (Figure 2). He received supportive treatment with intravenous hydration and intravenous metronidazole empirically. The stool culture was negative for infectious disease; hence, the antibiotic was discontinued.

He subsequently underwent a capsule endoscopy of the small bowels, which revealed severe inflammation characterized by erythema, edema, and aphthous ulceration in the ileum in a patchy distribution, with areas of skipped lesions. The proximal and mid ileum are more heavily involved than the terminal ileum. The capsule endoscopy finding of severe inflammation of the small intestine with areas of the skipped lesion is highly suggestive of Crohn’s disease. Serologic, genetic, and inflammation marker diagnostic tests confirmed the diagnosis of Crohn's disease. After an extensive rheumatology workup to rule out polymyositis, dermatomyositis, Sjogren's syndrome, ANCA vasculitis, and Celiac disease, it was concluded that LCV and myositis were extra-intestinal manifestations of Crohn’s disease in our patient. The patient was continued on high-dose prednisolone, and his symptoms improved remarkably. The steroid dose was tapered down successfully to 30 mg, and the gastroenterologist and rheumatologist followed him up as an outpatient. One week later, he reported remission of symptoms. The steroid dose was a short taper-off and was stopped within three weeks as the acute flare was already in remission. The patient did not need to be on long-term corticosteroids, and disease-modifying agents for Crohn's disease were commenced for long-term management.

## Discussion

The presentation of Crohn’s disease can be highly varied and unpredictable. The prevalence is higher among Caucasians [8-10]. Our patient is a young Caucasian male presenting with cutaneous vasculitis and evidence of myositis (muscle pain, muscle weakness, and elevated CK). Females are more likely to develop this syndrome than their male counterparts, like other autoimmune disorders, and this is usually attributed to the double X chromosomes in females [8-11]. Some studies have not shown gender differences in the characteristics and progression of the disease [12,13]. The development of the disease in young people can be a marker of severity as they live with it for a more significant part of their lives, thus affecting their overall quality of life [9,10].

The pathophysiology of Crohn’s is largely unknown, but some genetic associations exist. Researchers have found that the NOD2/CARD15 gene mutations on chromosome 16 are highly implicated in the pathophysiology of these conditions [14-16]. These changes are associated with higher chances of developing granulomas within the gut, forming the basis of the pathologic process. Chronic Inflammatory cells releases cytokines leading to the development of crypt abbesses, mucosa inflammation with attempts at fibrosis causes scarring and transmural damage to the layers of the gut [1,10,15,16]. Our patient was found to have these early inflammatory changes in the small bowel mucosa with evidence of ulceration. Skip lesions are one of the hallmarks of this condition compared to ulcerative colitis, where they are absent [1,10]. This was found in our patient with extensive inflammation in the proximal and mid areas compared to the terminal part of the ileum.

LCV is a cutaneous vasculitis that involves the deposition of active immune complexes in the capillaries of blood vessels [7]. This presents as a diffusely palpable purpuric lesion commonly affecting the LE. LCV is a rare disease, and its prevalence in patients with Crohn’s disease has not been extensively researched. It can occur as the initial presentation of Crohn’s disease, as seen in our patient, and can occur during disease flare [17-19].

Myositis is the development of active inflammation of muscle fibers with the elaboration of muscle-specific enzymes [20]. Crohn’s disease may present with various types of muscle inflammation, which range from myositis to polymyositis and dermatomyositis [1,5,6]. Our patient presented with severe muscle pains and weakness with a negative autoimmune myositis panel. We observed elevated CK in the setting of elevated ANA. These may suggest the possibility of association with Crohn’s disease rather than a primary inflammatory muscle disorder coexisting with Crohn’s disease.

Interestingly, the patient presenting symptoms of muscle pains and cutaneous vasculitis improved remarkably following prednisolone use. During the management of Crohn’s disease, it is essential to treat active flares with steroids and taper off to avoid the complications of prolonged steroid use [8,10]. In some severe cases complicated with strictures and fistula, immunotherapy with monoclonal antibodies is the cornerstone of management [2,3,8,10]. We highlight these findings to reinforce the need to have a high index of suspicion when patients with non-specific GI symptoms present with cutaneous and musculoskeletal manifestations. Providers should consider early referral for specialist involvement to prevent complications that arise from delayed diagnosis.

## Conclusions

Extra-intestinal manifestations associated with Crohn’s disease are not uncommon and can occur preceding active Crohn's disease or in the setting of active flares of the disease. In patients presenting with cutaneous vasculitis and negative autoimmune myositis workup, GI symptoms should always raise suspicion of inflammatory bowel disease (Crohn’s disease). They can precede the overt manifestation of the disease.
